# Practitioner research readiness in public health: findings from three co-produced surveys within local authority practice settings in England

**DOI:** 10.1186/s12889-025-25581-0

**Published:** 2025-12-30

**Authors:** Tanya Wright, Jay Tavernor, Gulshan Tajuria, Katharine Rodenhurst, Susan Lloyd, Phillip Northfield, Claire McIver, Emma Sandbach, Orsolina I. Martino, Ngozika Jane Hemuka, Jason Copp, Antony Stewart, Ross Wilkie, Paul Campbell

**Affiliations:** 1 Department of Research and Innovation, Midlands Partnership University NHS Foundation Trust, St. George’s Hospital, Corporation Street, Stafford, ST16 3SR UK; 2 School of Medicine, Keele University, Keele, Staffordshire, ST5 5BG UK; 3 Health, Wellbeing & Prevention Directorate, Shropshire Council, Shirehall, Shrewsbury, SY2 6ND UK; 4 Staffordshire County Council, Public Health, Health & Care Directorate, One Staffordshire Place, Tipping Street, Stafford, ST16 2LP UK; 5 Public Health Directorate, Research and Intelligence, Sandwell Metropolitan Borough Council, Sandwell Council House, Freeth St, Oldbury, B69 3DE UK; 6 University of Staffordshire, Stoke-on-Trent, ST4 2DE UK; 7 NIHR Clinical Research Network, West Midlands, UK

**Keywords:** Survey, Research, Public health, Research capacity, Local authority, Training

## Abstract

**Background:**

Evidence-based practice in public health is essential for delivering appropriate and effective interventions to improve population health. Robust research underpins evidence-based public health, and it is therefore vital that local public health practitioners have sufficient knowledge and skills to apply research as well as undertake research within their locality.

**Methods:**

This study aimed to assess individual research skills, knowledge, and confidence, as well as organisational support for research. Three local authority public health teams in England administered an internal service evaluation survey to their staff. Each survey included both closed (quantitative) and open ended (qualitative) items and obtained demographic information. Data from each participating local authority team were combined within a secondary data analysis utilising a parallel mixed method approach, reporting descriptive statistics, and deriving qualitative themes.

**Results:**

Across the three participating teams a total of 228 practitioners were invited and 109 responded (48% response rate). Both quantitative and qualitative results showed a clear recognition of the value of research to public health practice, both in general (66% indicated relevance to their practice), and as a method to understand local population health needs. Practitioners also wished to have more research involvement (81% reported research should be part of their CPD) but reported a current low level of research engagement and activity (36% had reported previous involvement over the previous 3 years). Recognised barriers to research engagement included, at a practitioner level: a lack of research skills resulting in low research confidence; time and capacity; organisational barriers such as resource provision (e.g. training, scholarship); and a lack of awareness of existing support structures.

**Conclusions:**

Public health practitioners recognise the value of research to their practice but require greater support (both from within local authority structures and externally) to create the framework to enable sustainable evidence-based practice.

**Graphical Abstract:**

**Figure:**
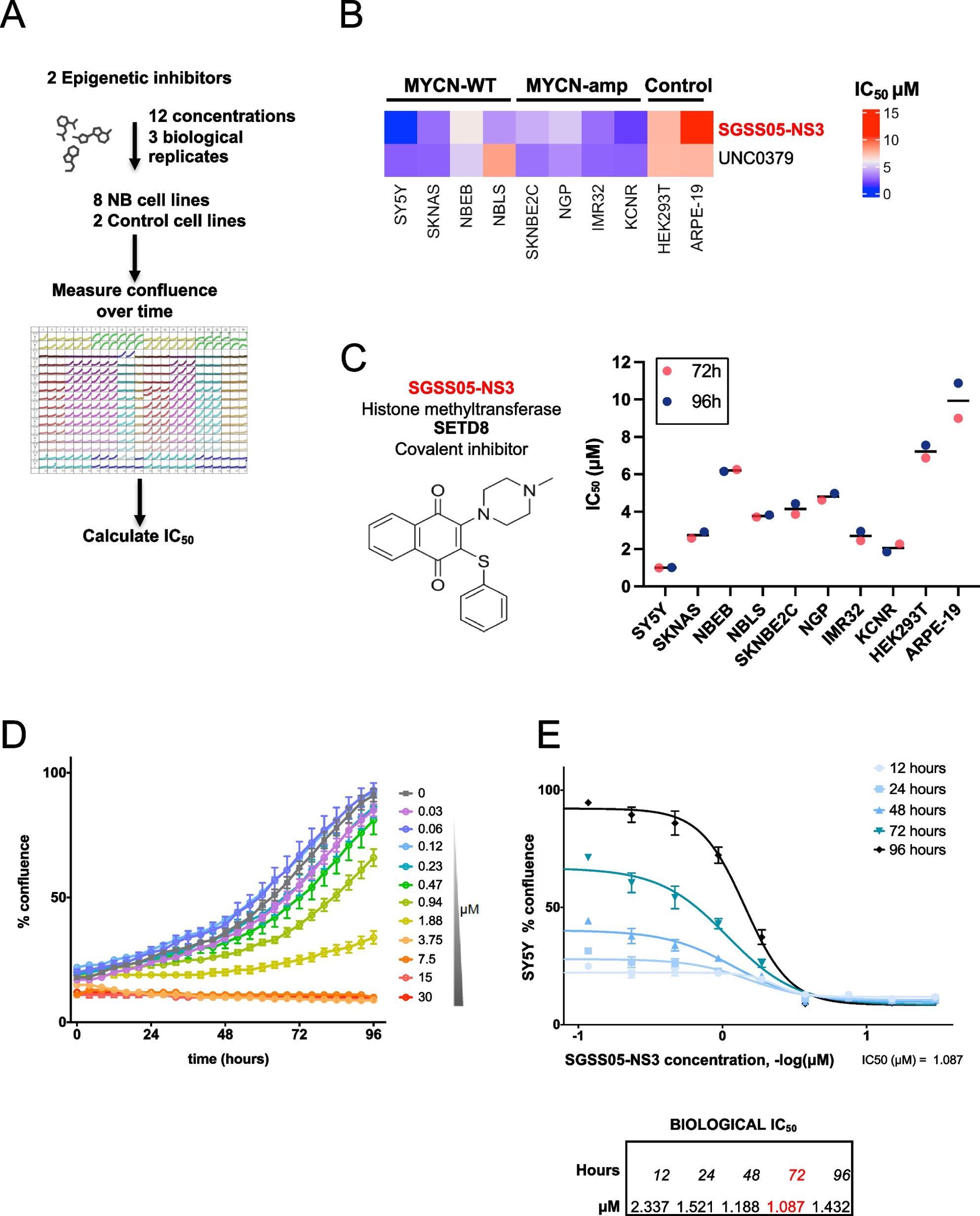
SCIENCE

**Supplementary Information:**

The online version contains supplementary material available at 10.1186/s12889-025-25581-0.

## Introduction

Evidence-Based Medicine (EBM) and Evidence-Based Practice (EBP) are well established within healthcare and involve the integration of current best research evidence, clinical and practice decision making, and patient values [1]. As such, it is important that those who apply EBM and EBP (via the systematic use of evidence, data, and information to improve clinical care) are aware and have knowledge of scientific and research-based principles [2–5]. EBM and EBP have demonstrated that the actual engagement of individual practitioners and their practice organisations in research (involvement and application) can lead to improvements in health outcomes and quality of care [6–8]. There is a long-established link in the United Kingdom (UK) between the National Health Service (NHS) and EBM and EBP [5, 9, 10]. Consequently, several organisations have developed to support EBM and EBP within the NHS such as the Cochrane Collaboration [11], and the National Institute for Clinical Excellence (NICE) [12]. Such evidence-based approaches are also applied to public health and the concept of Evidence Based Public Health (EBPH) is well established [13, 14]. Kohatsu et al. defines EBPH as “the integration of science-based interventions with community preferences to improve the health of populations” [4, p.419], taking the central concept of EBM and expanding it to include the health needs of a community or population. A key mechanism to underpin the application of EBPH is practitioner use of applied research within their locality to understand these population needs at a local level [13]. For example, Erwin et al.’s work in the USA on the development of Academic Health Departments (inclusive of practice and academics working together to address local public health needs) outlines the benefits from practice-based research which translates to health reform [15, 16]. Benefits of this translation of research to practice through the creation of evidenced-based interventions has demonstrated better quality information on what interventions work, coupled with a higher likelihood of success for evidence-based interventions and policies [17, 18].

While EBM and EBP in the UK are well established within the NHS, EBPH is less well established due in part to inherent structural differences as well as organisational changes over the last decade. Firstly, public health practitioners come from a wide diversity of backgrounds and therefore differences exist in their exposure to research and research training [18, 19]. Secondly, in 2013 Public Health in the UK transitioned from the remit of the NHS, into the remit of local authorities and Health and Wellbeing Boards [20]. A local authority is an elected governmental body responsible for the planning and provision of non-medical services (e.g. public health, social care, housing, education, transport) within a defined geographical area. This transition moved public health decision making, commissioning, and priority setting, to a local governmental level, at the same time as a significant reduction in funding [20]. Subsequent systems level research on the ability of UK local authorities to apply EBP (and by extension EBPH) has shown several challenges. Notable examples of the “needs” related to these challenges are to create engagement for staff to facilitate and action research, support for staff research skills development, and greater organisational research capacity building [21–24].

This current study aims to assess one key component of the overall challenges and needs required for EBPH, namely the “research readiness” at an individual practitioner level. Specifically, the assessment focus is on the local authority public health practitioner’s knowledge about research and current involvement in research, their research skills, abilities and confidence, as well as their knowledge/awareness of organisational structures to support research. This study forms part of a larger project called PRIDE (Public health Research Innovation anD Engagement), which is a programme of work led by a team from Midlands Partnership University NHS Foundation Trust to support research engagement for local authority public health practice. The surveys reported in this study were originally used by PRIDE to identify research knowledge gaps to inform a PRIDE led targeted research training programme for each involved practice team.

## Methods

### Study design, population, and setting

This study is a secondary data analysis of combined data from three cross sectional questionnaire-based surveys of public health practice research skills, research knowledge, research confidence, and research organisational support. The three surveys (all identical) were administered in 2023 to public health practitioners working in three separate local authority based public health teams within the Midlands region of England. More specifically, respondents were staff from Shropshire Council’s Health, Wellbeing and Prevention Directorate, Staffordshire County Council’s Public Health team, and Sandwell Metropolitan Borough Council’s Public Health team. Inclusion criteria for the identification of relevant staff were set by each participating public health team, meaning that each involved local authority public health team varied by size and by team definition (for example Shropshire public health team included social prescribers and environmental health practitioners, who in the other public health teams are situated elsewhere). There was a varied breakdown of the type of roles and duties of the respondents with roles titles such as Healthy Lives Advisors, Public Protection Officers, Programme Managers, Technical Officers, Public Health Consultants, and Social Prescribers and Environmental Health Practitioners (Shropshire only). Each team’s local authority region also differed by markers of health and deprivation: by health index score (HIS), which is a singular value with higher values corresponding to better physical, mental and social wellbeing [25] - Shropshire HIS 124.2, Staffordshire HIS 116.0, Sandwell HIS 85.2; and by deprivation index (based on several domains from the Office for National Statistics Census including; income, employment, education, health, crime and housing), with percentage not deprived in any dimension - Shropshire 50.8%, Staffordshire 50.3%, and Sandwell 37.9%.

### Survey procedure

**Fig. 1:**
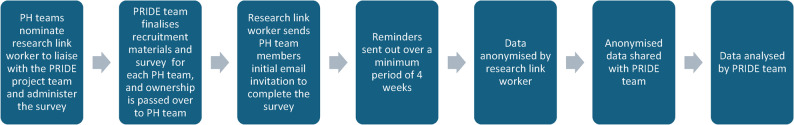
Survey procedure

The overarching PRIDE project had established engagement with each involved local authority public health team in advance of survey launch. This included initial meetings with practice team leads to support planned practice-based research activities. In our co-production model for the surveys, each engaged public health team nominated members of staff who would be research link workers (team-based survey leads) who would administer the survey to their respective team for their own team-based internal evaluation. Nominated individuals then worked with the PRIDE project team (JT, PC, TW, GT) on the processes to administer the survey (see Fig. 1). A team-based version of the survey (i.e. survey introduction and invitation to participate included wording specific to each team, though all questions remained the same) was created and a copy was passed over to each research link worker (giving full ownership to each team, i.e., PRIDE team members had no access thereafter). Participants were invited by the research link workers to complete the survey through email invitations. This involved an introductory email, participants information sheet, and instructions on how to complete the survey. Each team also operated a “reminder” system (via email) after survey launch to increase response rates, operating their recruitment for a period of at least 4 weeks. Once collected, data was exported internally to an Excel sheet and anonymised by the team-based survey lead, removing any personally identifiable information. The anonymised version was then shared with the PRIDE team for the purposes of firstly, co-creating an internal service evaluation report for each team separately, and secondly via agreement of the involved teams, combining the data to form a pooled analysis that forms the basis of this study.

### Measures

The measures used in this questionnaire survey were developed for this study (Research Readiness Questionnaire, Additional file 1 and Table 1) and are closely based on a previous version applied to assess research readiness within a Social Care workforce in England [26]. The term “Research Readiness” has received broad interpretations within the literature and can be seen as something that conceptualises organisational and structural support as well as readiness at an individual level [27]; for the purpose of use of the questionnaire in this study the focus is solely on the individual practitioners’ perceptions. Questionnaire items within the survey are based on previous literature and validated measures used to assess research capability, capacity and culture that can be applied within health, allied health and social care settings [28–32]. The questionnaire utilised a range of question types including dichotomous, categorical, and scale items, as well as qualitative open-ended items. The combination of both quantitative and qualitative items allows for a mixed-methods analysis approach increasing the richness, breadth, and depth, of the data collected [33]. Additionally, the questionnaire was designed to be brief to optimise response rate, taking between 10 and 15 min [34]. Table 1 details the domains, questions, and response categories for the questionnaire (for a copy of the RRQ see Additional file 1).

**Table 1:** Research readiness questionnaire items and response options

Domain	Question	Response options
Demographic	Age group	Categorical: Below 25, 25–29, 30–34, 35–39, 40–44, 45–49, 50–59, 60–69, Above 69
	Gender	Categorical: Female (incl trans women), Male (incl trans male), Non-binary, Don’t know, Prefer not to say, Other
	Current post	Free text
	Length of time in current post	Categorical: 0–3 years, 4–7 years, 8-15years, more than 15 years
	Full or part time	Categorical: Full time, Part time
	Current qualifications related to practice	Categorical: Certificate, Diploma, Bachelor’s, Master’s, Doctorate, Other
	Further study as part of public health career development	Categorical: None, Certificate, Diploma, Bachelor’s, Master’s, Doctorate, Other
	Years of practice	Categorical: 0–3 years, 4–7 years, 8–15 years, More than 15 years
	Size of public health team	Categorical: Sole practitioner, Below 10, 11–100, 101–1000, Above 1000
Existing Research Engagement	Are you now, or have you recently (past 3 years) been involved in research activity or research training?	Categorical: Yes, No, with open ended stem response for brief description if item response is yes
Importance/relevance of research	Do you think research should be a part of your own professional development?	Categorical: Yes, No
	How relevant do you feel research is in your current field of practice?	Likert scale: Not at all, Slightly, Moderately, Very, Extremely
Knowledge of and interest in research	Are you up to date with existing research literature and theory related to your field of practice?	Likert scale: Not at all, Slightly, Moderately, Very, Extremely For analysis purposes and ease of description the categories ‘not at all’ and ‘slightly’ were combined to form a single category and the categories ‘very’ and ‘extremely’ were also combined to form a single category. This resulted in 3 categories: ‘slightly’, ‘moderately’ and ‘very’.
	Have you an interest in conducting your own research, or including doing research as part of your overall career development?	
Organisational research support: Does your organisation have…	Adequate resources to support staff training	Categorical: Yes, No, Partly, Other
	Senior managers that support research	
	Ability to ensure staff career pathways are available in research	
	Ability to ensure organisational practice is guided by evidence	
	Software programs for analysing research data	
	Support with applications for research scholarships/degrees	
Research Skills – current confidence across a range of research skills	A. Finding relevant research literature? B. Critically appraising research literature? C. Designing a research study? D. Writing a research protocol? E. Collecting data e.g., surveys, interviews? F. Analysing qualitative research data? G. Analysing quantitative research data? H. Using computer data management and analysis programs (e.g., SPSS, STATA, NVIVO)? I. Writing a research report? J. Writing for publication in peer-reviewed journals? K. Submitting an ethics application? L. Applying for research funding?	Likert scale 1–5 with 1 being no confidence and 5 being full confidence For analysis purposes and ease of description 3 response categories were created: responses 1 and 2 were combined to form a ‘low confidence’ category, response 3 formed a ‘moderate confidence’ category, and responses 4 and 5 were combined to form a ‘high confidence’ category.
Questions about research	If you could ask a question to a leading public health researcher, what would it be?	Open ended free text response
	We would like to hear more about your thoughts on public health research and its relationship to you and your practice.	
	In your opinion what might be a key barrier or barriers to your involvement and engagement with research?	

### Analysis

Data was transferred to a Microsoft Excel 365 (version 2310) spreadsheet and quantitative descriptive analysis was conducted using IBM SPSS Statistics (version 27). Quantitative response items were analysed using descriptive percentage proportions for categorised or scaled responses. All data were included, and where applicable missing responses or ‘prefer not to say’ were included in the denominator value. Additionally, some response categories were collapsed. For example, grouping Likert responses or combining categories for ease of interpretation and to reduce opportunities for individual participant identification (Table 1). Responses from open-ended questions were analysed using a content analysis approach to systematically classify and describe meaning from the data. This approach was chosen due to the directive and limited nature of the data (i.e. singular question with no option for further exploration) and is therefore more restrictive than traditional qualitative inductive approaches [35–37]. Following previous methodology [36, 38], qualitative analysis of survey items considered individual responses as units to generate categories of meaning. Categories were then compared/contrasted across participant responses and further coding was applied to identify broader themes using an initial coding framework. Analysis was carried out independently by two researchers (TW, GT), who then combined their findings to develop the coding framework. Following that process the framework was shared with the wider team to agree consensus on the categories, codes, themes, with further rounds of interpretation.

A convergent parallel mixed approach was applied to both quantitative and qualitative data to inform the discussion and conclusions [39]. Integration of data was applied within the final interpretation stage of analysis using the qualitative data to identify a thematic framework, but also to inform on the quantitative descriptive results where applicable.

### Ethical approach

Each involved local authority public health team performed their survey as an internal service evaluation. Therefore, each team did not require formal ethical review or approval. Whilst ethical review was not required, each team applied ethical principles in the administration of their surveys. Prior to survey launch, all eligible participants were informed about the survey (e.g. purpose, content, data management and storage); that participation was entirely voluntary; that participants could withdraw at any time; that identifiable data would not be shared externally; and that findings would be completely anonymised and aggregated prior to external dissemination. Consent to take part was indicated by completion of the survey.

As this current study is a secondary data analysis of the anonymised pooled data there was no requirement for formal ethical review. However, consideration was given to ethical principles on the use of secondary data, such as the need to ensure that the research would do no harm, create prejudice or stigma, and avoid misinterpretation [40]. All participants were fully informed that the internal service evaluation was part of the wider engagement of each team with the PRIDE project (for the purpose of informing PRIDE on the research provision needs for each team) and that any data and findings shared externally would be anonymised). Furthermore, this study has been conducted in co-production with members of the public health teams as co-authors; this ensures that analysis was not done “on them” but “with them” to avoid misinterpretation of the data and findings. For a full description of this study’s ethical considerations please see Additional file 2, Appendix A.

## Quantitative results

### Sample characteristics

In total, 228 public health practitioners were invited to complete the survey and 109 responded resulting in a response rate of 47.8%. Table 2 details the demographic characteristics of the overall sample. In summary, the sample was predominantly female (69.7%; *n* = 76) and the age-group analysis showed the largest representation was from those over 50-years (39.4%; *n* = 43). Most (67.0%; *n* = 73) had been in their current post for 3 years or less, but over a third (37.6%; *n* = 41) had more than 15-years of experience and a majority of 71.6% (*n* = 78) worked full-time. Just under a third (31.2%; *n* = 34) had either a Master’s degree or a Doctorate. Job title data was collected but removed for anonymisation purposes, however the survey was administered to the full range of typical roles within the local authority public health teams, from Healthy Lives Advisors, Public Protection Officers, and Programme Managers to Technical Officers and Public Health Consultants, as well as those (within the Shropshire team) who may be deemed as complimentary to practice (Social Prescribers, Environmental Practitioners).

**Table 2:** Demographic characteristics

		Overall sample (*n* = 109)	
		Percent (%)	Number (n)
Gender	Male	27.5	30
	Female	69.7	76
	Other	2.8	3
Age Group	Under 29	7.3	8
	30–39	21.1	23
	40–49	32.1	35
	50–59	29.3	32
	60–69	10.1	11
Time in post (years)	0–3	67.0	73
	4–7	11.9	13
	8 or more years	21.1	23
Years of practice (years)	0–3	30.3	33
	4–7	14.7	16
	8–15	17.4	19
	> 15	37.6	41
Work pattern	Full time	71.6	78
	Part time	28.4	31
Qualifications	Diploma/certificate only	24.8	27
	Bachelor’s	24.8	27
	Bachelor’s plus other/diploma/certificate	8.3	9
	Master’s or Doctorate	31.2	34
	Other	11.0	12

### Existing research activity, research relevance to current field, research knowledge, and research interest

**Fig. 2:**
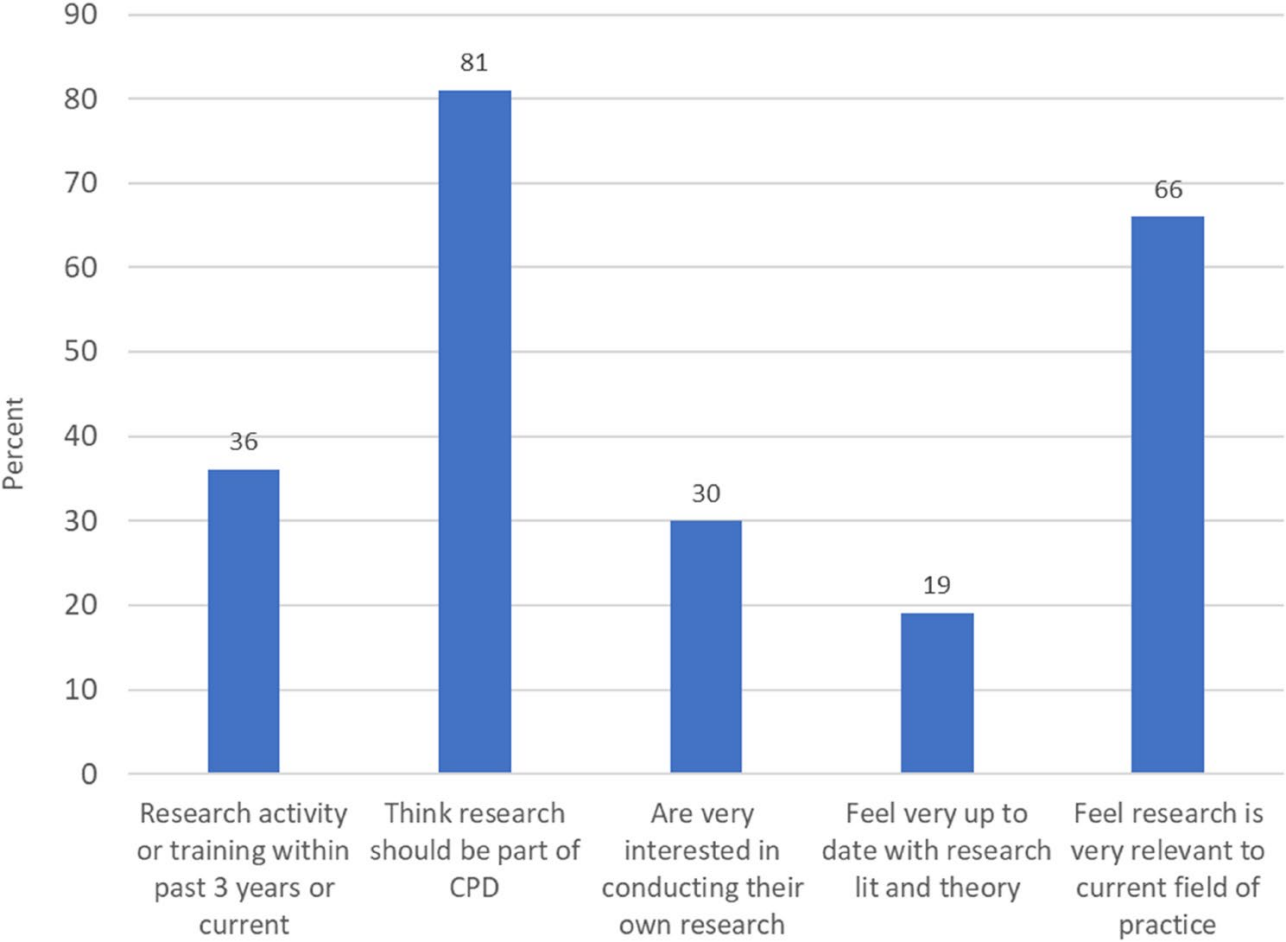
Existing research activity, knowledge, and interest

Findings on existing research activity, knowledge and engagement demonstrated that just over a third (35.8%; *n* = 39) had research activity or training within the past three years, and 80.7% (*n* = 88) thought that research should be part of their continuing professional development (CPD). While 66.1% (*n* = 72) felt that research was very relevant to their current field of practice, 19.3% (*n* = 21) felt very up to date with the research literature and theory in their particular field and just under a third (30.3%; *n* = 33) answered that they were very interested in conducting their own research. See Fig. 2.

### Research skills confidence

**Fig. 3:**
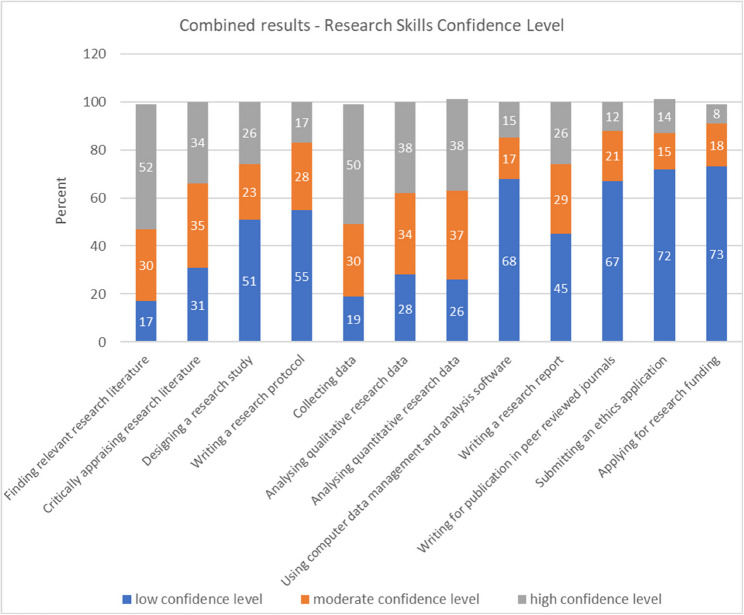
Research skills confidence level

There was a high level of confidence for 50% or more of participants in two skills areas: finding relevant research literature (52.3%; *n* = 57) and collecting data (50.5%; *n* = 55). Just over a third of participants had a high level of confidence in critically appraising research literature (33.9%; *n* = 37), analysing qualitative data (37.6%; *n* = 41), and analysing quantitative data (37.6%; *n* = 41), and just over a quarter had a high level of confidence in designing a research study (25.7%; *n* = 28), and in writing a research report (25.7%; *n* = 28). In the remaining skills, 11.9% (*n* = 13) were highly confident in writing for publication in peer reviewed journals and 8.3% (*n* = 9) were highly confident in applying for research funding. See Fig. 3 for more detail.

### Organisational resources and support

**Fig. 4:**
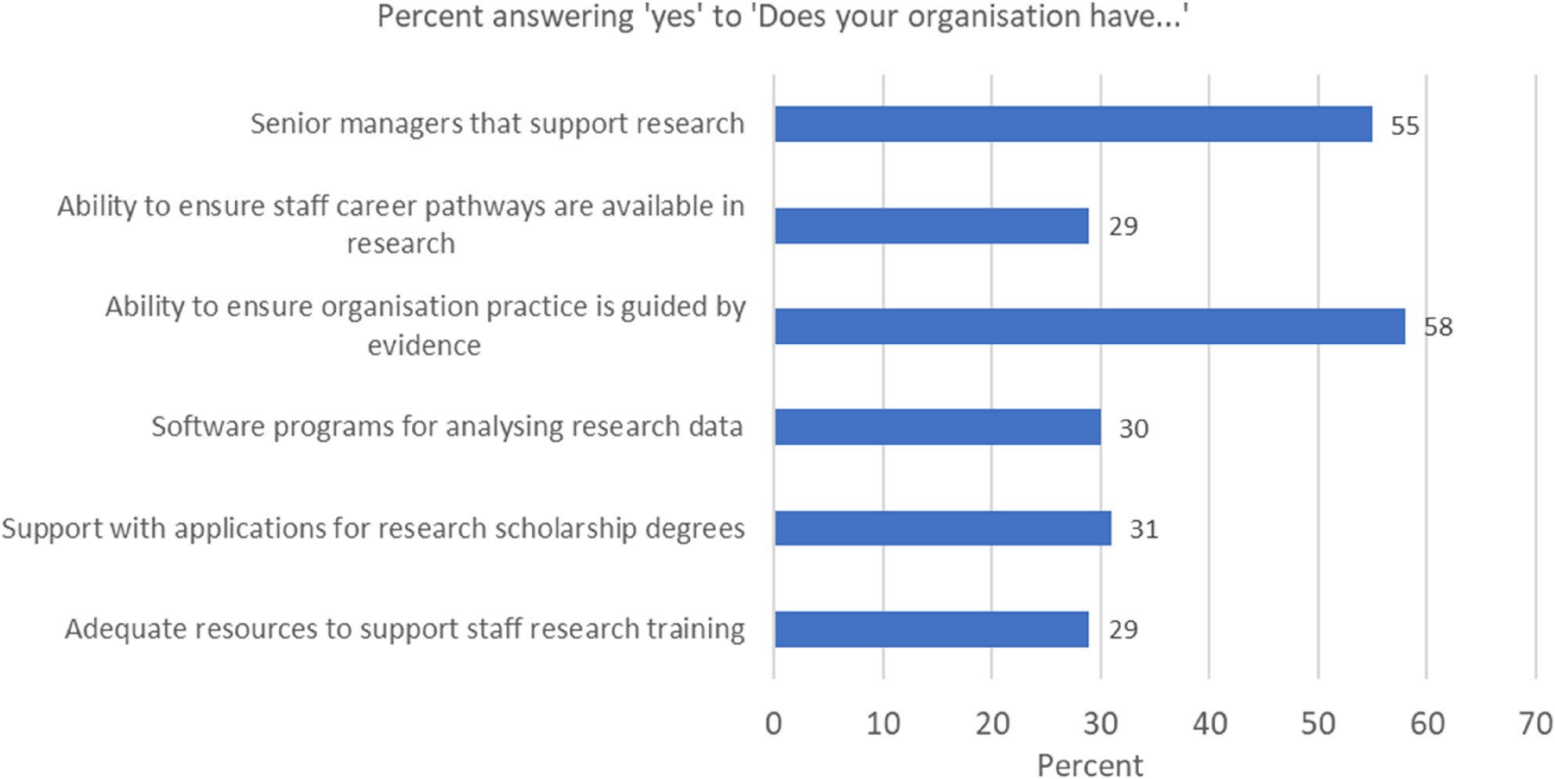
Organisational resources and support

Over half of participants agreed that their organisation has senior managers that support research and that their organisation has the ability to ensure organisational practice is guided by research. Agreement was selected by fewer than a third of participants for the remaining questions on ensuring availability of staff career pathways in research, providing software systems for data analysis, supporting with applications for research scholarship degrees and providing adequate resources to support staff research training (see Fig. 4).

### Qualitative results

In total, 58 (53%) participants responded to the first open ended question, 50 (46%) participants responded to the second open ended question, and 82 (75%) participants responded to the third open ended question (please refer to Table 1 for specific wording for these questions). Content analysis across the responses to the open-ended questions identified three main themes: “Significance of Research for Practitioners”; “Barriers to Conducting Research”; and “Research Interests”. Within these three overarching themes, several sub-themes were identified. For example, while responding to “Significance of Research for Practitioners”, participants acknowledged the strong links between research and public health practice in general (and the value of research therein), but also the significance of applying locally based research in providing needs-based support for local populations, as well as creating/facilitating a transparent process of undertaking research that can be accessible by the local population. Within the theme of “Barriers to Conducting Research”, several barriers were identified. A key barrier, highlighted by a majority of the respondents, was having time and capacity. Additional barriers were a lack of knowledge about potential research funding sources and funding application processes, a lack of knowledge of where to start or begin research and having a low level of research skills and experience leading to a sense of low confidence in the ability to undertake research. Further barriers to overall confidence were a perceived lack of organisational support to facilitate how research can feed into practice, and difficulty in comprehending and translating research to practice settings. Finally, within the theme of “Research Interests” participants suggested a wide range of research topics and priorities that they would wish to undertake including health in rural areas; health inequalities; ageing; global health; and long covid. A further overarching suggestion (which concords with the “Significance of Research for Practitioners” theme) was to regularly undertake more locally based comprehensive research that includes residents and member of the public, this action would underpin local based needs analysis to set intervention priorities. Table 3 details the qualitative results including themes, sub-themes with examples of supporting text.

**Table 3:** Qualitative findings

Theme	Sub-themes	Supporting text
Significance of research for practitioners’	Need-based support for local people Evidence based practice Value of research	*“…I am aware of national needs and targets and care about the local population; in that sense I am interested in how research can evidence not just needs*,* but also “what works”*,* in order to improve outcomes for local populations. Research has enabled professionals within public health to see what local needs are*,* and where they are*,* which in theory enables professionals to address those particular issues…” 1P12* *“…Our role [Public health practitioner] has evolved from epidemiological research and we now look at how everyday life*,* housing conditions*,* working conditions*,* the food we eat (from farm to fork) is all based upon research. We cannot stand still*,* and we need to look at PH challenges in the 21 st Century and address these accordingly through research” 1P29* *“I think it [research] is a gap in our current practice and is something we need to get better at embedding e.g. an evidence-based approach to all that we do*,* considering how we measure our impact*,* how we target our most in-need communities etc. We do this in part*,* but not always in a systematic way - and rarely with a true research focus. We are very poor at sharing our learning*,* adding to the evidence-base and celebrating our achievements/innovation” 2P10* *“How do we ensure research and data collated is clear and accessible for our population to understand?” 3P5*
Barriers to involve and engage with research	Resources (time and funding) Lack of skills Lack of confidence and knowledge on where to start Lack of support Lack of awareness and opportunity Lack of comprehensible research	*“Public Health departments are often stretched thin*,* with people filling multiple roles*,* so time would be a key issue” 1P10* *“Never had the funds to run research at a local level to get a meaningful sample and the packages eg SPSS or STATA or something similar to analyse the data” 3P46* *“There are also issues in applying for external funding*,* as funder’s deadlines do not always align to internal approval processes” 3P17* *“My main thoughts are around skills and maintaining them. I used to have good data analysis skills*,* but as I haven’t done that role for years*,* they are no longer very good…” 2P9* *“Confidence that i am going about it in the right way and that findings will actually be used and not wasted” 2P6* *“Main barrier is time*,* but also I haven’t done research for a long time so would really need some refresher training and guidance on how to go about it” 3P6* *“I am currently unsure where to start*,* unsure where to get training or what teams to reach out to in order to pursue this further. I have reached out in the past*,* but I have not really been pointed in the right direction*,* more to just online sites where I would love more guidance to get into public health research specifically in [Name]” 1P2* *“We need more support in our day-to-day practice to ensure that the research is given the proper level of priority as workloads often dictate a lesser focus on research that would be appropriate” 2P5* *“Not been asked to participate in research even though research is happening in the department” 3P33* *“…identifying areas that lend them self to research in the Local Authority is difficult as so much of our work is enabling work as opposed to designing and leading specific projects” 2P9* *“While the PH directorate has a strong culture of evidence-informed practice*,* this is not reflected across the whole council*,* and this can make it very difficult to incorporate research activity into timelines” 3P17* *“Not all professionals will understand research in depth*,* it would be good to have articles written in simpler layman terms. The knowledge can then be applied by community groups or organisations” 3P40*
Research interests	Research in health in rural areas Health inequalities Healthy Ageing Global health Long covid Comprehensive research	*“How can we evidence the positive and negative impacts of living in a rural area on health outcomes?” 1P12* *“Health inequalities and early mortality from poor housing is widely known*,* with initiatives*,* such as additional insulation not really addressing all of the adverse health effects. What other*,* more drastic*,* approaches would you like to see?” 1P13* *“…how could it be improved the healthy ageing based on some factors like deprivation/diverse communities/ethnicities/language barrier/religion etc” 3P49“* *…how easy might it be to access professionals who have carried out or are carrying out relevant research in other countries - other countries will be operating different initiatives and models and I would want to ascertain level of success and consider demographics to consider whether their different approaches might have a positive impact here…” 1P14* *“Is there a body of evidence indicating that Covid and/or Covid vaccinations are contributing to a rise in people suffering long term Covid symptoms*,* vestibular issues and problems with balance and proprioception and mini strokes” 1P35* *“I think it would be useful for us to really be able to dig deeper into the behaviour/practice and outcomes of our local residents as it would really inform our future commissioning and help to identify our local needs*,* particularly qualitative research and engaging with those who use (or may use) our services rather than just looking at quantitative information and national guidance” 2P13*

## Discussion

### Summary of results

This study has reported on the assessment of public health practitioners’ knowledge about research, their current involvement in research, their research skills, abilities and confidence, as well as knowledge and awareness of organisational support for research within three local authority public health practice teams in England. Results indicate that a majority of practitioners feel that research should be included in their CPD, and over two-thirds believe that research and EBPH are relevant (and intrinsic) to their current field of practice. However, paradoxically, only a minority of public health practitioners have current or recent research involvement, have an interest in conducting their own research, or feel up to date with current literature and theory.

### Comparison with previous literature

Our findings are broadly in line with international and UK based previous literature (including systematic reviews) that has examined research capability, capacity and culture across various allied health and social care practice settings [26, 28, 41–46]. Similar broad conclusions can be drawn from the wider literature on practitioner’s recognition of the value of research, but that a lack of research-based skills, resources, time, and organisational support significantly hamper opportunities to engage and undertake research (either individually or collectively). Focusing on previous research specific to UK local authority settings (where public health practice occurs) there are also similar findings. A recent qualitative exploratory study [47] looked at the use of research evidence across social care and public health and reported key barriers that include access, research timeliness, local applicability, and competence in finding and interpreting evidence, all in broad accord to this study’s findings. Similarly, Sabey et al.’s [43] needs assessment of research training undertaken across both health and local authority organisations from the West of England reported that evidence-based practice is generally accepted but faces challenges due to practical constraints such as staffing levels and capacity. They found that priorities for workforce were for statutory and mandatory training (rather than research), and that most organisations lacked a systematic approach for research training. Our findings reiterate these broader conclusions and extend this knowledge base further by detailing specific information on local authority public health practice.

### Interpretation and implications

The survey results, used independently within each public health team, have informed on specific research needs, which have, in turn, highlighted several courses of action for the overarching PRIDE project. Taken collectively the surveys reported within this study give an overview of practitioner research readiness drawn from a varied sample of practitioner roles within three different practice local authority regions in England. These findings may offer some representativeness to other local authority public health practice teams, though further research would be required to substantiate this conclusion. Clear implications have been identified, with overwhelming recognition of the value and place of research within practice (81% of respondents), and the relevance of research to practice (66% of respondents). In terms of “research readiness” two forms of understanding can be drawn from these results based on the distinction between having the ability to apply research knowledge for practice and the ability to actually undertake research activity in practice. The results show a high perception (66%) that research is relevant to practice, whilst also showing a relatively low level (30%) of practitioner interest in conducting their own research. These overall perceptions can be mapped to the assessment of practitioner confidence across a range of skills; with an increased level in the use of research skills (e.g. finding literature, critically appraising literature, and analysing data) compared with the skills required for the processes to “do” research (e.g. writing a research proposal, finding suitable funding, study design, ethics, using analysis software, writing for publication). This suggests that practitioners are relatively more confident (and ready) to apply research knowledge, but less “ready” in the skills needed to undertake research activity. These findings support an element of practitioner “readiness” in the application of EBPH but are also suggestive of a need for much more support to enable research that is relevant and applicable at a local level [20, 48], a point highlighted further within the qualitative evidence where practitioners recognise the need for research to understand “local needs” and evaluate the impact of locally based interventions. Research, and the ability of practice to use research, is a cornerstone of policy development and advocacy [13, 49]. All of these points are considered key areas of competence in the UK Faculty of Public Health Curriculum [50] and are a feature of the ten essential public health practice operations as prescribed by the World Health Organisation [51]. Furthermore, examination of accepted models of EBPH suggest that whilst the use of the best available evidence is a core component, so is the need to understand population characteristics, needs, and values (due in part to the growing recognition of demographic inequity), aligned with resources required by practice to undertake this approach [13, 52–54].

Consideration of the identified barriers indicates potential solutions to the greater uptake of EBPH. The PRIDE project, which organised the surveys for our involved public health teams, employed collaboration between practice and academia as a core operating principle. This facilitated the provision of “hands on” research advice and guidance, signposting to relevant research skills training, and joint engagement to support practitioner research career and project funding opportunities. This key point of collaboration between practice and academia has been shown to be effective in the development of locally based public health research activity previously, particularly a model which involves a “research” lead within the practice organisation [45, 55, 56].

Addressing the barriers to research engagement from an individual practitioner perspective is only part of a wider issue of required organisational and structural support. One key barrier identified is practitioner capacity and time. Results from the questions on organisational resources and support show that whilst a majority (55%) of practitioners reported being supported by senior management, there was a deficiency in the supply of supportive structures such as adequate resources to support research skills development, and a lack of support to research career development (e.g. development of research career pathways, access to research scholarship). This latter point on research career development seems to be exacerbated by a lack of knowledge on existing provision within intra-organisational supportive structures in the UK. For example the funded research career pathways open to local authority-based professionals (at pre-doctoral, doctoral, and advanced levels) as provided by the National Institute of Health and Care Research (NIHR) as well as a host of other avenues for engagement (e.g. NIHR School for Public Health Research, NIHR Short Placement Awards for Collaboration, NIHR Incubators, NIHR Public Health Research for Health Consortiums, and NIHR Health Determinants Research Collaborations which are 5 year programmes to increase local authority research capacity and capability). Whilst many supportive structures are now in place, they are relatively new in operation within local authority settings [47], and it may be of interest in the forthcoming years to see if such broad-based approaches lead to increased local authority public health practice engagement with research.

### Strengths and limitations

There are several strengths associated with this study. To our knowledge this study is the first to provide detailed information regarding the assessment of research skills, research knowledge, research confidence, and research organisational support within local authority-based public health in England. In addition, the survey responses were obtained from a wide range of roles within public health practice and included practitioners with a range of years of experience. Furthermore, the included practice teams operate within different local authority regions which have differing levels of local health needs, contexts, and settings (e.g. very rural in Shropshire, to heavy urban in Sandwell). The response rate to the survey was 47.8%, which can be considered higher than many other surveys of this type (e.g. Sabey et al., 2019 at 36%, Friesen and Comino, 2017 at 26%). Finally, the combination of both quantitative and qualitative data adds richness and breadth to our findings. There are some weaknesses to the study. We cannot rule out the possibility of response bias and social desirability bias. Those who responded to the survey invitation may have been more likely to have an interest in research and have previous involvement with research; therefore, our findings may well include over estimations of research readiness. Similarly, questions regarding organisational support and resources, may have been influenced by the perception that more ‘positive’ responses would be ‘expected’ as the surveys were internal to each team. Another limitation is the cross-sectional design, as this only captures a single moment and is unable to capture trends or developments over time. Finally, the qualitative element of the study was limited to open ended free text questions, and thus there was no capacity to further probe and explore further as would be the case in the use of interview methodology [57].

In conclusion, this mixed methods survey has demonstrated that despite a recognition of the relevance and value of research and a desire for research to be included in CPD, there are relatively low levels of actual research engagement, activity, and interest within local authority public health practice settings. Significant gaps identified are a lack of research skills, confidence in the application of research, and organisational capacity to resource and support research activities. Findings suggest that greater organisational supportive structures (from within and outside of local authorities) are required to support practitioner research engagement.

## Supplementary Information

Supplementary Material 1. Research Readiness Questionnaire. Copy of the questionnaire used by each local authority public health team for their internal service evaluation of research readiness.

Supplementary Material 2. Ethical Considerations. Full description of the ethical considerations for this study. 

## Data Availability

The anonymised datasets used and/or analysed during the current study are available from the corresponding author on reasonable request.

## Abbreviations

CPD: Continuing Professional Development
EBM: Evidence Based Medicine
EBP: Evidence Based Practice
EBPH: Evidence Based Public Health
NHS: National Health Service
NIHR: National Institute of Health and Care Research
PRIDE: Public Health Research Innovation and Engagement
UK: United Kingdom
