# Supplementary material for: Practitioner research readiness in public health: findings from three co-produced surveys within local authority practice settings in England
Source: BMC Public Health. 2025 Dec 30;25:4383. doi: 10.1186/s12889-025-25581-0 (PMC12755017; doi:10.1186/s12889-025-25581-0)
Supplement: Supplementary file 1 — Supplementary Material 1. Research Readiness Questionnaire. Copy of the questionnaire used by each local authority public health team for their internal service evaluation of research readiness. [file 12889_2025_25581_MOESM1_ESM.pdf]

Research Readiness Questionnaire – Public Health

Section 1 - Participant information

**1.**	**Name:**
**2.**	**Work email:**
**3.**	**Job title:**
**4.**	**Organisation:**

Section 2 - Demographics

5. **Your age?** *Please choose from the options below*

Below 25
25 – 29
30 – 34
35 – 39
40 – 44
45 – 49
50 – 59
60 – 69
Above 69

6. **Your gender?** *Please choose to which gender you most identify*

Female (including trans woman)
Male (including trans man)
Non-binary
Don’t know
Prefer not to respond
Other (please specify)

7. **Current post?** *Please describe in the box below*

8. **Length time in your current post?** *Please choose from the options below*

0 – 3 years
4 – 7 years
8 – 15 years
Above 15 years

9. **Is your current post full-time or part time?** *Please choose from the options below*

Full time
Part time

10. **Your current qualifications related to your practice?** *Please choose from the options below*

Certificate
Diploma
Bachelor
Masters
Doctorate
Other (please specify)

11. **Are you undertaking further study** as part of your public health practice career development at present? *Please choose from the options below*

None
Certificate
Diploma
Bachelor
Masters
Doctorate
Other (please specify)

12. **Years of practice?** *Please choose from the options below*

0 – 3 years
4 – 7 years
8 – 15 years
Above 15 years

13. **The size of your public health team?** *Please choose from the options below*

Sole practitioner
Below 10
11 – 100
101 – 1000
Above 1000

Section 3 - Existing research engagement

14. **Are you now or have recently (in the past 3 years) been involved in research activity or research training?** *Please choose from the options below*

Yes
No

15. **If Yes – Please give a brief description of what research activity or training you have been involved in.**

Section 4 - Importance/Relevance of research

16. **Do you think research should be part of your own professional development?** *Please choose from the options below*

Yes
No

17 Please briefly share the reasons for why you selected your response in the above question: ***Please describe in the text box below***

18. **How relevant do you feel research is in your current field of practice?** *Please choose from the options below*

Not at all
Slightly
Moderately
Very
Extremely

19. **Please briefly share your reasons why you selected your response in the above question?** *Please describe in the box below*

Section 5 - Knowledge/Interest of/in research

20. **Are you up to date with existing research literature and theory related to your field of practice?** *Please choose from the options below*

Not at all
Slightly
Moderately
Very
Extremely

21. **Have you an interest in conducting your own research, or including doing research as part of your overall career development?** *Please choose from the options below*

Not at all
Slightly
Moderately
Very
Extremely

Section 6 - Organisational research support

This section asks about your current knowledge about research support within your existing organisation (e.g. local authority)

22. **Has adequate resources to support staff research training?** *Please choose from the options below*

Yes
No
Partly
Other (please specify)

23. **Has senior managers that support research?** *Please choose from the options below*

Yes
No
Partly
Other (please specify)

24. **Ensures staff career pathways are available in research**? *Please choose from the options below*

Yes
No
Partly
Other (please specify)

25. Ensures organisation practice is guided by evidence? *Please choose from the options below*

Yes
No
Partly
Other (please specify)

26. Has software programs for analysing research data? *Please choose from the options below*

Yes
No
Partly
Other (please specify)

27. Supports applications for research scholarships/ degrees? *Please choose from the options below*

Yes
No
Partly
Other (please specify)

Section 7 - Research skills

28. The following questions ask about your current confidence across a range of research-based skills

Response scale is scored 1 to 7 with 1 indicating no confidence and 7 equating to full confidence.

A) Finding relevant research literature?

1	2	3	4	5	6	7

B) Critically appraising research literature?

1	2	3	4	5	6	7

C) Designing a research study?

1	2	3	4	5	6	7

D) Writing a research protocol?

1	2	3	4	5	6	7

E) Collecting data e.g. surveys, interviews?

1	2	3	4	5	6	7

F) Analysing qualitative research data?

1	2	3	4	5	6	7

G) Analysing quantitative research data?

1	2	3	4	5	6	7

H) Using computer data management and analysis programs (e.g. SPSS, STATA, NVIVO)?

1	2	3	4	5	6	7

I) Writing a research report?

1	2	3	4	5	6	7

J) Writing for publication in peer-reviewed journals?

1	2	3	4	5	6	7

K) Submitting an ethics application?

1	2	3	4	5	6	7

L) Applying for research funding?

1	2	3	4	5	6	7

Section 8 - Your questions about research

29. **If you could ask a question to a leading public health researcher, what would it be?** *Please describe below*

30. **We would like to hear more about your thoughts on public health research and its relationship to you and your practice, please use the following text box to give any further information….**

31. **In your opinion what might be a key barrier or barriers to your involvement and engagement with research?**
