# Supplementary material for: Practitioner research readiness in public health: findings from three co-produced surveys within local authority practice settings in England
Source: BMC Public Health. 2025 Dec 30;25:4383. doi: 10.1186/s12889-025-25581-0 (PMC12755017; doi:10.1186/s12889-025-25581-0)
Supplement: Supplementary file 2 — Supplementary Material 2. Ethical Considerations. Full description of the ethical considerations for this study. [file 12889_2025_25581_MOESM2_ESM.pdf]

Additional file R2

Appendix A: Ethical Considerations

This current study involved a two-stage process. The first stage involved agreement from all participating local authority public health teams (as named within the paper) to take part in the PRIDE (Public health Research Innovation anD Engagement) project. This engagement project (i.e. not research) had the aim of creating research engagement within each practice team. This included linking up each practitioner team with relevant academics and researchers (from within PRIDE) to create a platform to discuss locally based research needs with each team independently (crafting research ideas, research funding applications, and support to individual practitioners for their own research career development). Part of PRIDE's objectives was to assess each participating practice teams' research awareness, engagement, skills, and confidence (research readiness). This assessment was carried out to tailor bespoke research support provision for each team. Each participating team agreed to undertake their own internal service evaluation using our survey template and proposed method (this was initiated to ensure that each team followed good practice). The survey was run by each participating team, with only fully anonymised results shared with the PRIDE team, as agreed by each participating team and as per participant consent to take part (i.e. that anonymised data would be shared). This facilitated the production of an individual team-based report on research readiness to inform research support provision per team. These above actions were not considered as "research" and therefore did not qualify for consideration of research ethics (local or otherwise).

At stage two, subsequent to the above activity commencing, there was discussion between the PRIDE team with all participating practice teams of the potential value of pooling the anonymised data (as collected at stage one) for a secondary data analysis. It was recognised this would be a unique opportunity to illustrate a wider practice perspective on research readiness. At stage two there was agreement from each participating local authority public health team to use this anonymised data as held by PRIDE to produce (via co-production) a pooled analysis (the findings of this paper).

This study has followed relevant guidance (ESRC Framework for Research Ethics) that outlines specific criteria for secondary data analysis across three categories; i) data not sensitive/minimal risk of disclosure, ii) data protected by legislation (e.g. census, admin data), iii) data where combination/linkage may increase identifiability. Our assessment indicated that category (i) was appropriate (data contains no identifiers, participants were not vulnerable, content of the data is not sensitive, and no new data was collected). Based on this assessment ethical review was not required.

This approach is reflective of previous recent UK research which has taken a similar position on the non-requirement of ethical review in the case of analysis of data from non-research sources such as service evaluations/anonymised data (*Leary et al. 2024. Why are healthcare*

professionals leaving NHS roles? A secondary analysis of routinely collected data. Human Resources for Health, 22(1), 65; Wakefield et al. 2022. Social work and social care: Mapping workforce engagement, relevance, experience and interest in research. The British Journal of Social Work, 52(4), 2291-2311; Moule et al. 2016. Practical guidance on undertaking a service evaluation. Nursing Standard, 30(45)). In addition, as outlined in the paper, whilst ethical review was not required the study did apply ethical principles within the data collection at stage one (informed consent, voluntary participation, right to withdraw, anonymisation of data prior to sharing with PRIDE team). In addition, at stage two further consideration was given to ethical principles, such as the need to ensure that the research would do no harm, create prejudice or stigma, and avoid misinterpretation, and this was done via co-creation with members of the participating public health practice teams (analysis and interpretation as well as co-author contributions).
